# From Caves to the Savannah, the Mitogenome History of Modern Lions (*Panthera leo*) and Their Ancestors

**DOI:** 10.3390/ijms25105193

**Published:** 2024-05-10

**Authors:** Camilla Broggini, Marta Cavallini, Isabella Vanetti, Jackie Abell, Giorgio Binelli, Gianluca Lombardo

**Affiliations:** 1 Wildlife Research Unit (UIRCP-UCO), University of Cordoba, 14071 Córdoba, Spain; z62brbrc@uco.es; 2 Department of Biotechnology and Life Sciences (DBSV), University of Insubria, 21100 Varese, Italy; m.cavallini1@uninsubria.it (M.C.); isabella.vanetti@uninsubria.it (I.V.); giorgio.binelli@uninsubria.it (G.B.); 3 Centre for Agroecology, Water and Resilience, Coventry University, Coventry CV8 3LG, UK; jackie.abell2@coventry.ac.uk

**Keywords:** lion phylogeny, *Panthera leo*, *Panthera atrox*, *Panthera spelaea* mitogenome, big cat evolution, cave lion, molecular phylogeny, evolutionary biology

## Abstract

Lions (*Panthera leo*) play a crucial ecological role in shaping and maintaining fragile ecosystems within Africa. Conservation efforts should focus on genetic variability within wild populations when considering reintroduction attempts. We studied two groups of lions from two conservation sites located in Zambia and Zimbabwe to determine their genetic make-up, information that is usually unknown to the sites. In this study, we analysed 17 specimens for *cytb* and seven microsatellite markers to ascertain family relationships and genetic diversity previously obtained by observational studies. We then produced a standardised haplogroup phylogeny using all available entire mitogenomes, as well as calculating a revised molecular clock. The modern lion lineage diverged ~151 kya and was divided into two subspecies, both containing three distinct haplogroups. We confirm that *Panthera leo persica* is not a subspecies, but rather a haplogroup of the northern *P.l. leo* that exited Africa at least ~31 kya. The progenitor to all lions existed ~1.2 Mya, possibly in SE Africa, and later exited Africa and split into the two cave lion lineages ~175 kya. Species demography is correlated to major climactic events. We now have a detailed phylogeny of lion evolution and an idea of their conservation status given the threat of climate change.

## 1. Introduction

The lion (*Panthera leo*) is a carnivorous mammal belonging to the feline family which plays an important role in the food chain, regulating the numbers of the most dominant herbivore species, besides being one of the most charismatic animals on Earth. Lions appeared in Tanzania between 3.46 and 1.2 Mya [1,2], becoming the most widespread carnivorous mammal on Earth, spanning Africa, Europe, and Asia (Figure S1A). In recent times, however, lions have been restricted almost exclusively to Africa, where, because of the anthropic impact upon landscape, whose main and most evident effect is habitat disruption and/or fragmentation, large populations of these felines survive only in reserves and large parks of southern Africa [3].

The International Union for Conservation of Nature (IUCN) in 2016 recognized two existing subspecies of lion: the African lion, *Panthera leo leo* (Linnaeus, 1758), located throughout Sub-Saharan Africa with the exception of the rainforests areas; and the Asian lion, *Panthera leo persica* (Meyer, 1826), which is recovering from near extinction and is now present only in the Gir forest in India in low numbers [4]. In 2017, the two subspecies were described as the North African lion, *Panthera leo leo*, north of the East African rift, and *Panthera leo melanochaita*, south of the rift. However, no specification for *Panthera leo persica* was given [5].

In addition to modern lions, we know of three other extinct species of lions: *Panthera spelaea* or cave lion, distributed in Eurasia and Beringia; *Panthera atrox* or American cave lion, found in North America; and *Panthera fossilis*, also found in Eurasia. The latter, however, has historically been classified as a separate species due to its different stratigraphic position and morphology. However, the species has recently been deemed an ancestral *P. spelaea* chronospecies, since the differences in morphology were mainly in size and the two overlapped temporarily [6]. Cave lions and modern lions have been found to have diverged during the Pleistocene between 1.2 and 2.9 Mya, calculated using both fossil and molecular estimates [7,8].

These results indicate that cave lions and modern lions are indeed different species, with the former further split into two main haplogroups which diverged ~578 kya. The American cave lion, on the other hand, diverged from a population of *P. spelaea* around ~165 kya, which became separated by the Laurentide ice sheets; this lineage is recognised today as *Panthera atrox* and its mitochondrial origins can be dated to ~81 kya [9].

The IUCN Red List of Threatened Species currently lists lions as “Vulnerable” [10], giving an estimate of closer to 23,000 wild lions living in Africa and now extinct in 26 different African countries [11]. Contrary to the IUCN definition, molecular analyses [12] have shown that there are at least three distinct populations of lions located in as many different areas of the continent: the population located in North Africa–Asia, that of Southern Africa, and that of Central Africa. The level of genetic differentiation between lions coming from the three macro geographical areas (Northern Africa, Eastern Africa, and Central-West Africa) is quite high, as shown by the *F*_ST_ values between 0.064 and 0.396; the Indian population is very well differentiated from the African ones, having an *F*_ST_ value of 0.736 [13].

Fortunately, in African lions, no evidence of genetic erosion appears. In a study of 15 African populations based both on mtDNA and nuclear microsatellites [3], the nucleotide diversity (*π*) value at the mtDNA level was 0.102, estimated on 87 sequences of 1454 bp, and the polymorphism values were close to 100%, while *H*_e_ values were between 0.41 and 0.70. In the same work, an Asian population was also used as a comparison, for which the levels of genetic variability were much lower, with a polymorphism value of 25% and *H*_e_ equal to 0.13. Asiatic lions have low genetic diversity compared to African lions, having discovered a total absence of variability for each locus of the DNA sequences studied compared to the moderate levels of variation for the same loci analysed in African lions [14].

This scenario must, however, be maintained, if not reversed, because we can otherwise expect, in a few generations, a reduction of gene flow as a consequence of the fragmentation of the populations and both genetic drift and inbreeding to slowly erode the gene pool of today’s lions as a consequence of the serious decline in the number of specimens. Therefore, in managing the remaining populations it is vital to minimise the loss of genetic variability: transferring animals to nearby areas should also ideally imitate the natural gene flow. This is an important concern when lion populations are involved—the genetic tool should become more and more relevant for planning sound conservation strategies, being complementary to the eco-ethological aspects of reintroduction projects.

The aims of this work are, therefore, multiple:

1. To verify, by analysis of the mtDNA, the family structure established by field observations and study the phylogenetic relationships of our specimens, through a comparison with the information available in the literature.
2. To study, by means of microsatellite markers, the variability and differentiation of two lion populations for their management and reintroduction into the wild.
3. To confirm the species and subspecies status of *Panthera leo*, *P. spelaea*, and *P. atrox*, and determine the divergence times of their main haplogroups.
4. To produce a standardised phylogeny and develop a molecular clock for lions.

## 2. Results

### 2.1. Phylogeography of Modern and Cave Lions

#### 2.1.1. Species Determination via *Cytb* Sequence

The phylogenetic analyses for *cytb* revealed that all 277 mitogenomes clustered into three main branches based on the alleged species, with *Panthera spelaea* (*n* = 56) having three main sub-haplogroups (S1a, S1b and S1c) and being a sister clade to *Panthera atrox* (*n* = 11), which has two main sub-haplogroups (X1 and X2).

**Figure 1:**
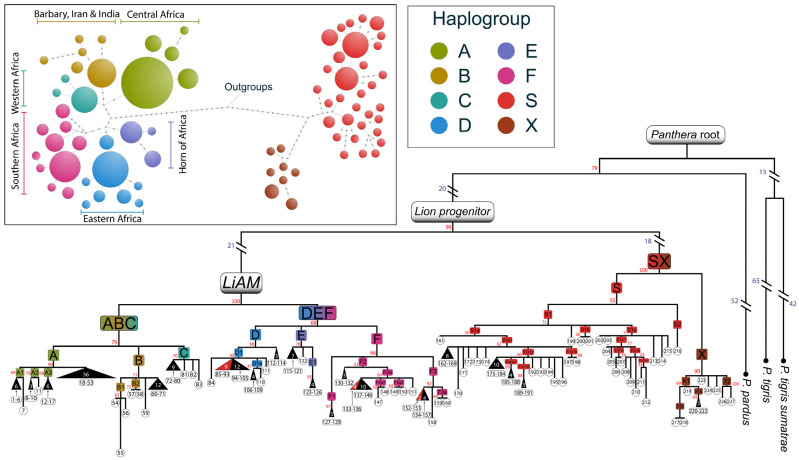
Schematic maximum parsimony *cytb* phylogeny. Haplogroups are represented as coloured squares and sample IDs are written in the circles. Triangles represent a group of individuals with the same haplotype, triangle bases are proportional to the number of mitogenomes. Different colours were assigned to major haplogroups (squares). Branch length is proportional to the number of nucleotide substitutions, blue values indicate the number of nucleotide substitutions of out-of-scale branches, and red values indicate bootstrap values. Inset: haplogroup phylogeny of *cytb* sequences. Sizes of circles are proportional to the number of *cytb* sequences, with the smallest circle representing one individual. Dots on branches represent intermediate haplotypes.

Moreover, *Panthera leo* (*n* = 160) clustered into six main haplogroups, named A through F. These form two main clusters (Figure 1) and correspond to two of the three postulated lion subspecies: the first, *P.l. leo*, encompasses haplogroups A (Central Africa), B (Barbary, Iran and India), and C (Western Africa); while the second, *P.l. melanochaita*, encompasses haplogroups D (Eastern Africa), E (Horn of Africa), and F (Southern Africa). The nucleotide diversity values within the six haplogroups ranged from 0.03 to 0.33 (lowest in C and highest in F), while the values between them confirmed the two subspecies split with values ranging from 0.80 to 1.12 (BD and AF, respectively). The third described subspecies, *P.l. persica*, had low nucleotide diversity values compared to the closely related haplogroups A and C (Table S1), suggesting the same subspecies. All modern lion haplogroups derive from a common female ancestor denominated LiAM (Lion Ancestral Mitogenome). Moreover, our analysis also revealed that the 15 sampled specimens share three haplotypes distributed into three sub-haplogroups (D1 and F2a and F3).

#### 2.1.2. Entire Mitogenomes Reveal Lion Evolutionary History

**Figure 2:**
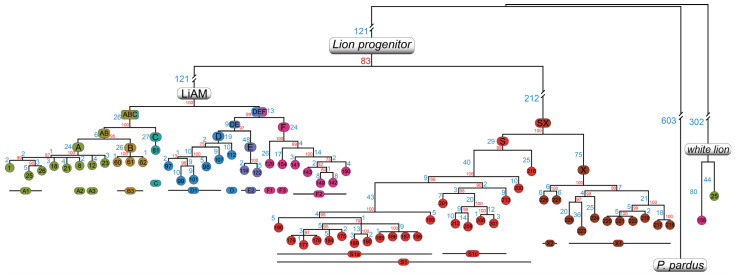
Schematic maximum parsimony mtDNA coding region phylogeny. Different colours were assigned only to major haplogroups and are consistent with other figures. Main haplogroups are indicated at the node while sub-haplogroups are indicated below. Numbered circles refer to the same individuals as Figure 1. Numbers on branches indicate the number of nucleotide substitutions. Red values indicate bootstrap values.

The study of the entire mitogenome revealed new haplogroups which were determined by mutations outside the *cytb* region (Figure 2); these were A4 (DRC), B3 (India specific, *Panthera leo persica*), and E2 (Somalia and India). The nucleotide diversity was found to be greatest at the control region (Figure S3) and signs of positive selection were seen in the non-synonymous/synonymous mutations ratio in the *ATP8* gene for *P. atrox* and *P. spelaea*, and to a lesser extent also in *P. leo*, and positive selection for the *ND3* gene was seen in *P. atrox* (Figure S4).

Haplogroups A and B shared a common ancestor (node AB), indicating that haplogroup C split off early and reached western Africa, then A and B split, giving rise to a lion population that stayed in central Africa (haplogroup A) and one that migrated north (haplogroup B). On the other hand, haplogroup F split from node DE and moved to southern Africa, with haplogroups D and E splitting later and colonising eastern Africa and the Horn of Africa, respectively. Furthermore, samples #25 and #158 were found in Genbank as “white lion” sequences, and although they clustered correctly in haplogroups F and A in *cytb* analysis, they appeared to be as divergent to lions as panthers and tigers are when considering the entire mitogenome.

#### 2.1.3. Times of Population Expansion

**Figure 3:**
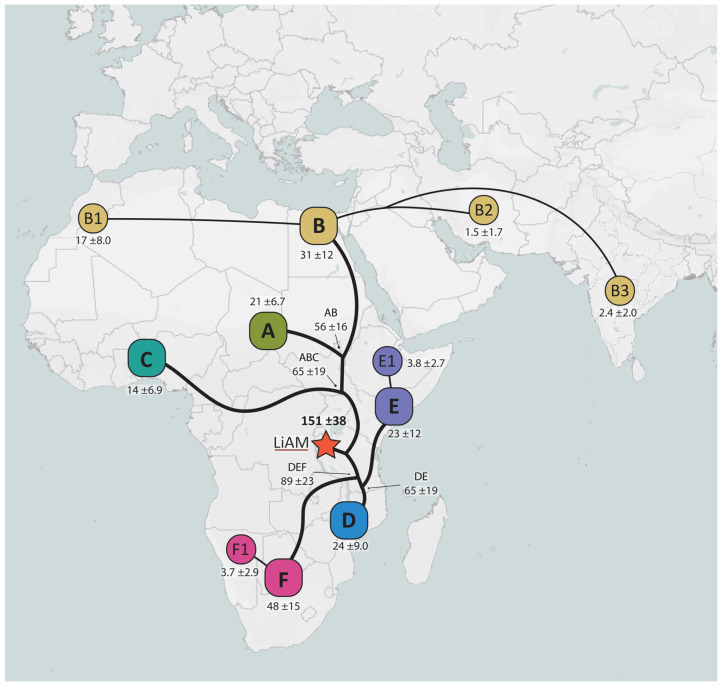
Modern lion population spread through time. Map is based on *cytb* divergence times obtained with ML molecular clock and Bayesian analysis. Squares indicate main haplogroups while circles indicate sub-haplogroups. Colours are consistent with other Figures. Thick lines indicate main haplogroup expansions and thinner lines indicate sub-haplogroup expansions. Numbers above or below haplogroups indicate the coalescence times in thousands of years ago (kya) with their respective standard deviation. Numbers at nodes indicate separation times between lineages (kya). The red star indicates the lion ancestral mitogenome (LiAM), or mitochondrial eve.

**Figure 4:**
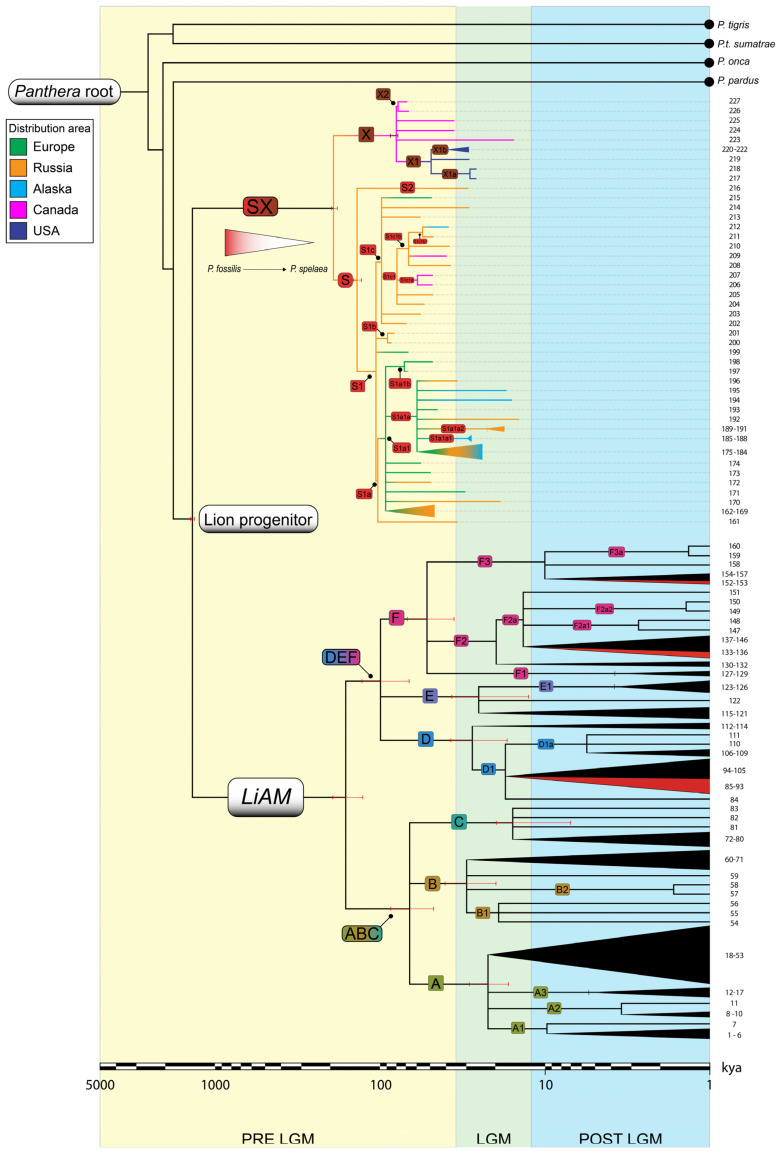
Bayesian *cytb* phylogeny with coalescence times. Haplogroups and sub-haplogroups are represented as coloured squares, colours are consistent with other Figures. Triangles represent individuals with the same haplotype and bases are proportional to the number of mitogenomes. The gradient arrow below branch SX indicates the substitution of *Panthera fossilis* with its chronospecies *Panthera spelaea*. The timeline (log_10_) at the bottom refers to the Bayesian coalescence times in thousands of years ago (kya) and is split into three periods (coloured background): pre-Last Glacial Maximum (LGM), LGM, and post-LGM. For cave lions, branch colours indicate area of sampling.

The age estimates for all *cytb* nodes were obtained using Bayesian analysis and a strict molecular clock previously calculated via the maximum likelihood (ML) method (Table S2). We calculated that the split between all lion species lineages occurred 1264 ± 33 kya, which is in the range of previous works [7] and fossil data; we named this individual “Lion Progenitor”. Then, according to our calculations, the female lioness carrying the ancestral modern lion mitogenome (LiAM) lived around 151 ± 38 kya, possibly in southern Africa as the oldest haplogroups are found here (Figure 3). This node is also the time of the first phylogenetic split which gave rise to the two subspecies: *P.l. leo*, including haplogroups A, B, and C, which all moved north and west (65 ± 19 kya); and *P.l. melanochaita*, haplogroups D, E, and F, which moved south and east (89 ± 23 kya). Haplogroup A originated in central Africa 21 ± 6.7 kya ago and encompasses four main sub haplogroups (A1–A4), all dated between 1.3 and 9.4 kya. Haplogroup B moved to northern Africa, possibly following the Red Sea coast around 31 ± 12 kya, splitting on the way into three main sub-haplogroups: B1, found in Barbary and dated 17 ± 8.0 kya; B2, found in Iran and dated 1.5 ± 1.7 kya; and B3, found in India and dated 2.3 ± 2.0 kya. The distribution of B sub-haplogroups show an out-of-Africa dispersion pattern. Haplogroup C is found in western Africa and originated 14 ± 7.0 kya. Haplogroup D originated in eastern Africa 24 ± 9.0 kya and comprises nine of our 15 new samples, all of which belong to the main sub-haplogroup, D1, dated 15 ± 4.8 kya. Haplogroup E instead moved towards the Horn of Africa and its origins are dated to 22 ± 10 kya; there are two sub-haplogroups present: E1, dated 3.8 ± 2.7 kya; and E2, dated 3.1 ± 2.7 kya. Finally, Haplogroup F is found in southern Africa and is dated to 48 ± 15 kya; it encompasses three main sub haplogroups: F1, dated 3.7 ± 2.9 kya; F2, which comprises four of our samples and was dated 20 ± 7.3 kya; and F3, which comprises two of our samples and was dated 16 ± 7.1 kya (Figure 4).

We estimate that the cave lion lineage originated around 175 ± 8.5 kya and further split into *P. spelaea* 131 ± 3.5 kya and *P. atrox* 81 ± 8.5 kya. All major sub-haplogroups are pre-Last Glacial Maximum (LGM), with most of them becoming extinct before the LGM. Very few linages survived during the LGM; these are S1a1a with its two sub-haplogroups in Russia and Alaska, and X1 and its sub-haplogroups in current day USA.

#### 2.1.4. Demography of Lions over Time

**Figure 5:**
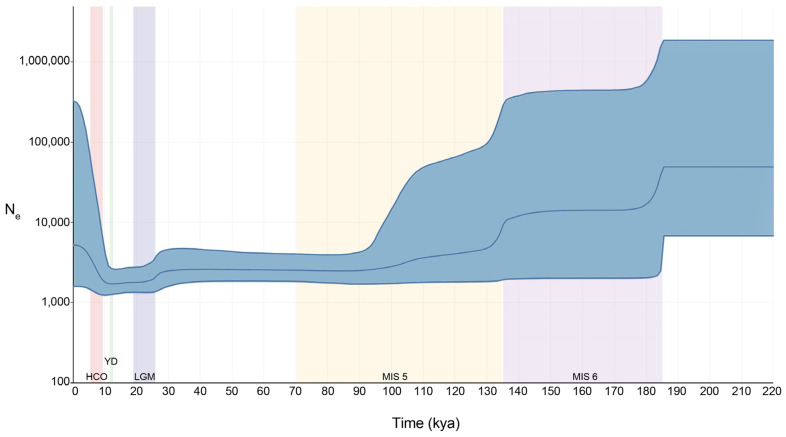
Bayesian Skyline Plot of lion mitogenomes. The plot considers all *cytb* samples listed in Supplementary Table S3. These include *Panthera leo*, *Panthera spelaea*, and *Panthera atrox*. The blue line within the shaded area indicates the median estimate of the effective population size and the blue shading shows the 95% highest posterior density limits. The time axis is limited to 220 kya, beyond which the curve remains flat. Background colours represent: HCO, Holocene Climactic Optimum; YD, Younger Dryas; LGM, Last Glacial Maximum; MIS5, Marine Isotope Stage 5; MIS6, Marine Isotope Stage 6.

The Bayesian skyline plot (Figure 5) shows the change in effective population size (*N*_e_) over time for all lions (Haplogroups A, S, and X). The first demographic event occurred ~185 kya at the beginning of the Marine Isotope Stage 6 (MIS6). The decrease in the effective population size (*N*_e_) is sharp and in concordance with the beginning of the glaciation period followed by a plateau for roughly ~50 kya. The second demographic event occurred at the beginning of the interglacial MIS5, and though it was not as sharp as the first decrease, *N*_e_ slowly reduced throughout the whole era with a smaller reduction around ~109 kya, corresponding to the MIS5d glacial peak. The final demographic decrease occurred at the beginning of the LGM and corresponds to the extinction of most *spelaea* and *atrox* individuals. The final demographic event is a sharp increase in *N*_e_ occurring after the Younger Dryas (YD) at the beginning of the Holocene Climactic Optimum (HCO) ~10 kya.

### 2.2. Nuclear Genetic Variability

In addition to *cytb* sequencing, we genotyped 17 lions from the field for seven microsatellite loci and added their genotype data to an already published dataset [3] for a combined total of 139 samples genotyped using seven microsatellite markers (Table S4). We are aware of possible differences in peak analysis when studying genotype data obtained in different manners, but our mitochondrial data further corroborate our results, suggesting that the nuclear microsatellite analysis was correctly performed. The Polymorphism Information Content (PIC) was calculated for all SSRs and ranged between 0.7 and 0.9, indicating largely informative sites [15]. The expected (*H*_e_) and observed (*H*_o_) heterozygosity values were calculated for each population (Table S5), for the total population, and for each species. The *H*_e_ values varied from 0.14 (India) to 0.70 (Zimbabwe and Zambia) with an average value equal to 0.56, while the values of *H*_o_ appeared to be relatively higher for the single populations, where they were between 0.17 (India) and 0.83 (Benin), with an average of 0.61. The inbreeding coefficient, *F*_IS_, values were below 0.3, indicating an excess of heterozygotes for all populations.

Differentiation tests were performed using Genetix v.4.05.2 (Belkhir et al., Montpellier, France) [16]. Weir and Cockerham’s estimator of *F*_ST_ [17] was obtained overall and for each population pair (Table S6). The null hypothesis of identical allelic distribution among all populations was tested by means of a permutation test both at the overall level for the populations considered, and at a more specific level by carrying out differentiation tests between pairs of populations. All *F*_ST_ values were significantly different from zero. What emerges from this data is a medium-to-high total differentiation value (total *F*_ST_), equal to 0.29, with a confidence interval that ranges between 0.25 and 0.32. At the inter-population level, the highest values of differentiation were found to be those relating to the Indian population. The lowest values were those of Central Africa, while our studied populations (Zimbabwe and Zambia) were found to have intermediate values, which can also be clearly seen from the PCoA (Figure S6).

**Figure 6:**
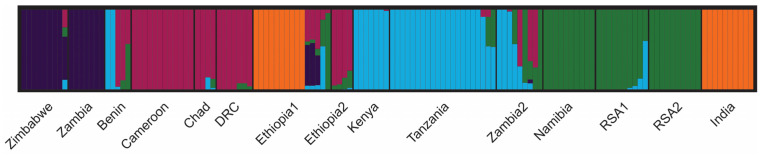
Genetic structure of the studied lion populations as inferred by Bayesian clustering of SSR genotypes at *K* = 5. Each vertical bar represents a single individual and the proportion of membership to a genetic pool is indicated by the different colours.

The software structure v.2.3.4 (Pritchard, Stanford, CA, USA) was used to investigate the genetic composition of the population, and we identified the most likely number of ancestral gene pools to be *K* = 5 (Figure S7) using Evanno’s Δ*K* method [18]. In Table S7, we report the probability that a given population may have originated from the same ancestral population. Finally, we used clumpp v.1.1.2 (Rosenberg, Stanford, CA, USA) and distruct v.1.1 (Rosenberg, Stanford, CA, USA) on the structure results to obtain the barplot of the average run for each *K* value (Figure 6). The populations of Cameroon, Chad, and DRC (represented by magenta bars); the populations of Kenya and Tanzania (represented by blue bars); the populations of Namibia, RSA1, and RSA2 (represented by green bars); and the Indian population (orange bars) all belong to single differentiated gene pools. Regarding the Ethiopian populations, the first group clustered with India while the second one clustered with the group of central African countries. The lions from both Benin and Zambia 2, on the other hand, have three main origins. Two lions from Benin were found to share the same proportion of ancestral genome with central African lions, another two had east African origins most likely due to reintroduction policies, and one lion had admixed origins. The Zambia 2 lions also showed these two different origins with the addition of two lions having south African origins.

## 3. Discussion

### 3.1. A New Phylogeny for Lions

Our analysis found three main sub-haplogroups of *Panthera spelaea* and two in *Panthera atrox*; the first shows a west-to-east expansion with the most recent sub-haplogroups appearing in in Asia and Alaska. The latter, on the other hand, most likely originated from a *P. spelaea* population in Canada or Alaska which separated from the mainland Eurasia population, as can be seen by the low number of mutations between haplogroups S and X and from the *cytb* nucleotide diversity value of 1.74 ± 0.17 (Table S1). Values below 2% are indicative of a within-species status [19]; therefore, as far as the *cytb* is concerned, we can consider these either as sister species or *P. atrox* as a subspecies of *P. spelaea*. Moreover, *Panthera leo* clustered into six main haplogroups as previously postulated [20,21,22]. These two main subclades follow a geographic distribution pattern and have nucleotide diversity values (0.80–1.12) compatible with the existence of two lion subspecies, *P.l. leo* [23] haplogroups A, B, and C; and *P.l. melanochaita* [12] haplogroups D, E, and F. This, however, cannot be said for *P.l. persica* (haplogroup B). This postulated subspecies was found to have a difference of between 0.43 and 0.54% with *P.l. leo*, far below the nucleotide difference observed with the other confirmed subspecies. This, together with the few private mutations present in haplogroup B phylogeny (with respect to haplogroups D, E, and F, for example), is not enough to consider *P.l. persica* a separate subspecies.

The analysis of the complete mitogenomes was consistent with the *cytb*, and, even with a reduced number of individuals, confirmed the main topography of the tree while further subdividing a few haplogroups only present in the *cytb* tree at the macro level. From this tree we programmed a script for mtPhyl v. 5.003 (Eltsov & Volodko, Novosibirsk, Russia) to automatically classify whole mtDNAs (Figure S2) into the correct haplogroups and draw out the detailed phylogeny; this could be useful for future classification and conservation studies. The produced tree further explained the relationships between sub-haplogroups of the two main postulated subspecies of modern lions. Once again, *P.l. persica* was found to have too few mutations when compared to the close A and C to be considered a subspecies. This was backed by the resolved phylogeny of the entire mitogenome, as haplogroups B and A shared a more recent common ancestor than haplogroup C. A further in-depth analysis of sequences deposited as “white lion” (#25 & 158) showed that roughly half the mitogenome had very few mutations (from the control region to the *ND1* gene; from half of the *ND4* gene to the control region), thus classifying the samples as *P.l. leo* and therefore consistent with the *cytb* analysis as the gene was included in the range. The other half of the mitogenome had a rather large and unaccountable number of mutations for the two samples to be considered a lion, most likely due to the presence of many nuclear mitochondrial DNA (NUMTs) in the sequence, as has been previously observed [24,25]. The white lion, like the Indian lion, cannot be classified as a subspecies, as one individual is a *P.l. leo* and the other is *P.l. melanochaita*. We therefore confirm the subspecies status of two out of the three postulated subspecies of *Panthera leo*.

Judging by the fossil record, the presence of close species, and our coalescence times, the origin of the genus *Panthera* occurred somewhere in Asia [26]. Later, an ancestor to panthers and the Lion Progenitor migrated to Africa at least 3.6–3.8 Mya in concordance with the fossil record [27]. Their ancestral lines then split around 1.8 Mya (Figure S5). Like previously said, the ancestor to all lions lived around 1.2 Mya, most likely in modern day Tanzania. According to our model, the lion lineages then split into modern and cave lions; the latter, judging by the fossil record, exited Africa and reached Eurasia. Here, it became smaller, as seen by several morphological traits [6] until at least 175 kya, when, from this study, the main lineage of *Panthera spelaea-atrox* (node SX) arose, indicating the continuity and subsequently the substitution of *P. fossilis* with *P. spelaea*, confirming the chronospecies status of the former. Here, they settled into Europe and Russia first, as reflected in the most ancient individuals, then into Beringia and later into northern America. This west-to-east movement can be seen in Haplogroup S1a1a2, ranging from the Urals all the way to the Bering strait and dated at 52 kya, but individuals within the group form younger nodes while moving east towards the Bering strait.

Cave lions reached North America on at least two different occasions, the first during the MIS6 around the aforementioned 175 kya of the two cave lions split, which is in concordance with a previous study [9]. An ancestor to both *spelaea* and *atrox* reached North America via the Beringian land bridge and diverged into the two species giving rise to the haplogroup X progenitor 81 kya. There is no trace of *spelaea* here until much later, indicating the extinction of the progenitors in Beringia. Later, a wave of *Panthera spelaea* (individuals from sub-haplogroup S1a1a) reached Alaska around 92 kya. This sub-haplogroup further diverged to form S1a1a1, which is only found in eastern Alaska and was dated to 39 kya. This split from a Beringia population around 59 kya. Another two sub-haplogroups of *spelaea* can be found in North America (S1c1a and S1c1b) dated to around 80 kya and went extinct ~30 kya. Finally, sub-haplogroup S1a1b can only be found in eastern Russia and Austria and is dated to 69 kya. Due to the limited number of whole mitogenomes and their incomplete presence in all haplogroups, time estimates calculated with whole mtDNA sometimes differ from the values obtained via *cytb* analysis. Both estimates are reported in Table S2.

The two main modern lion lineages (ABC and DEF) all originated pre-LGM, with the initial split between the two subspecies occurring 151 kya, most likely in the African Rift Valley, with the geographical barrier restricting gene flow (Figure 4). The LGM drove the further differentiation of the main haplogroups as all but haplogroup F were dated to this event (14–24 kya) probably due to refugia with restricted gene flow occurring, as can be seen in climatological data [28]. Most sub-haplogroups then emerged during the post-LGM with the climate being milder. The Indian individuals have a rather large coalescence date span (3.5–35 kya) due to there being few whole mtDNAs and a lack of sub-haplogroups which increases the error in dating. Though deep node ages between *cytb* and whole mtDNA appear to be very different due to the date overestimation of the shorter *cytb* sequence, coalescent ages of more recent nodes and leaves are similar (Table S2). Once again, the dates confute the existence of a *P.l. persica* subspecies, though radiation times are consistent with an out-of-Africa migration to the Arabian Peninsula post-LGM.

### 3.2. Climate Change and Lion Diversification

The demography of lions confirms the effects of climate change on the history of these species. Considering standard deviation error, the drop in the effective population size, *N*_e_, reflects the separation dates of *P.l. spelaea* and *P.l. atrox*, probably brought on by the climate change occurring during the beginning of the MIS6. The second population decrease at the beginning of the MIS5 occurred at the time of the formation of haplogroup S and LiAM, once again linking the change in climate to a speciation event. The final demographic decrease occurred at the beginning of the LGM and corresponds to the extinction of most *spelaea* and *atrox* individuals and is within previously obtained dates [29]. The warming temperatures at the end of the Younger Dryas brought on a large radiation of *Panthera leo* populations, with many sub-haplogroups diverging at this stage. From the BSP data we can see how climate change played an important role in shaping the demography of the three species as well as driving speciation and extinction events. Here, we confirm that, as previously speculated [30], the demographic drop and subsequent extinction of cave lions in Eurasia occurred at the same time as the disappearance of prey species [31,32,33].

### 3.3. Genetic Variability and Conservation

Nuclear data showed healthy populations with an average heterozygosity value of 0.56. Indian populations harboured the lowest value (0.14), which is likely due to two bottlenecks/founder effects which occurred during the population’s history. The first bottleneck/founder effect occurred when haplogroup B split from haplogroup A and followed a northward path to Egypt, possibly following the Nile River. The second event occurred at the split between haplogroups B2 and B3, with the latter reaching India. All other major haplogroups in fact have much higher heterozygosity values (0.46–0.70), and therefore a higher genetic variability; this can be attributed to some level of gene flow occurring between the other populations having remained in Africa.

### 3.4. Bayesian Analysis and Population Structure

An interesting picture emerged from the analysis of nuclear variability by means of SSRs. We identified five ancestral gene pools of origin. We are aware that we have not been working on a “true” population, but rather on a random sample of lions from a given geographic region. However, we found out that our sample behaved as a real population, as its most evident feature is the common inferred origin of the Zimbabwe and Zambia lions, which was found to be different from all others, thus suggesting that the studied lions may represent a novel gene pool. In fact, there are several findings from our work that could support this hypothesis. First, the genetic variability was remarkably like that estimated in another work [3], and this showed the expected heterozygosity values for 15 populations across Africa ranging between 0.41 and 0.70. Considering that the *H*_e_ values obtained in this work for the Zimbabwe and Zambia samples were 0.70 and 0.59, respectively, we can start to say that at least considering the populations under study, there are no signs of genetic erosion. Moreover, the populations from Cameroon, Chad, DRC, and Ethiopia2 appear to belong to the same cluster, as well as populations from Namibia, RSA-A, and RSA-B, and populations from Kenya and Tanzania. The genetic differentiation values between our lions and those coming from other macro geographical areas were found to be overall medium-high, with a total *F*_ST_ equal to 0.29. This value perfectly falls within previously obtained ranges [3] of 0.06–0.40. These values could be explained by the presence of natural structures such as the Rift Valley or the African rainforests that would have acted as barriers to maintain the differences between West/Central African lions and those in the southern regions. These finding are in concordance with our mitochondrial analysis, as our six haplogroups corresponded to five of the ancestral gene pools. With the absence of genotyped western African samples, the sixth potential ancestral gene pool is absent, which would be corresponding to haplogroup C. The only populations not to show a definite structure were Benin, Zambia 2, and, at least in part, Ethiopia 1. The latter showed some common origin with the Indian gene pool, leading us to guess that at least a few lions were imported from Asia or, as mitochondrial analysis suggests, haplogroups B and A have a common origin and therefore individuals in central Africa, such as those in Ethiopia, could still harbour the same genetic profile of Indian samples.

## 4. Materials and Methods

### 4.1. Sample Collection

Samples were derived from lions from two different sites. First, we analysed lions from a pride of captive-bred origin, the Ngamo pride, owned and managed under ALERT (African Lion and Environmental Research Trust) and located 13 km outside of Gweru (Zimbabwe) within a fenced game reserve. The samples from the second group came from a sanctuary situated in the Mosi-oa-tunya National Park, Livingstone (Zambia). After the morphological identification of every lion, 17 faecal samples were collected during research sessions: in Table S8 information about sampling is reported, together with other geographical and time data. Once collected, the stool samples were immediately preserved in ethanol; DNA extraction was carried out using the Stool DNA Isolation Kit (Norgen Biotek, Thorold, ON, Canada) following the manufacturer’s instructions. Extracted DNA was visualised for integrity and quantified in a 0.8% agarose gel: 3 μL of each sample was loaded with 1 μL of loading dye. Integrity of DNA samples was verified by comparing them against a 1kb DNA Ladder (SibEnzyme, Novosibirsk, Russia).

### 4.2. Analysed Samples for Mitogenome Variation

A 718 bp region of the mitochondrial cytochrome b (*cytb*) gene was amplified and successfully sequenced in 15 individuals using PCR-based methods with locus-specific primers (Table S9) forward (2F) and reverse (4R) [3]. All amplifications were performed in 20 μL reaction volume containing 4 μL PCR buffer, 0.25 μM forward primer, 0.25 μM reverse primer, 12.8 μL water, 0.2 μL Taq Polymerase, and 0.2 μL of DNA extract. Amplification was performed on an Eppendorf AG 22331 Thermocycler. Samples were denatured at 94 °C for 4 min, PCR profiles consisted of 40 cycles as follows: 94 °C for 20 s, annealing temperature of 52 °C for 1 min and 72 °C for 1 min, with a final elongation period of 10 min at 72 °C. The results of the PCR were evaluated by electrophoresis; positive samples were sequenced using external service and data verification was carried out manually with BioEdit v.7.2.5 (Hall, Tampa, FL, USA) [34].

For *cytb* analysis, in addition to the 15 partial *cytb* sequences obtained in this study, 145 modern lions were included from GenBank (accession numbers can be found in Table S3), together with 56 Eurasian cave lions (*P. spelaea*) and 11 American cave lions (*P. atrox*) *cytb* sequences. Sequences were aligned by hand using MEGA v.11.0.13 (Tamura et al., Tokyo, Japan; Philadelphia, PA, USA) [35]. The complete 1040 bp sequence was artificially reconstructed for those individuals who had gaps in the sequence by using the lion reference mitogenome and taking into consideration haplogroup mutations; therefore, though incomplete, it was possible to assign individuals to haplogroups and allowed for the reconstruction of the *cytb* maternal phylogeny. Moreover, to test the validity of the *cytb* tree, 66 available entire mitogenomes were aligned (without control region or indels) for complete mtDNA analysis. Of these mtDNAs, 26 were from modern lions, 29 from Eurasian cave lions, and 11 from American cave lions.

The detailed mitochondrial DNA map (Figure S8) of Genbank sample NC028302 [36] was built using Proksee v.1.1.1 (Grant et al., Alberta, CA, Canada) [37] and mapped using GC Content (Window size 500bp, auto step size), GC Skew (Window size 500 bp, auto step size), and MITOS v.2.1.3 (Donath et al., Bonn, Germany); refseq 89 Metazoa, Genetic code 2: Vertebrate mitochondrial [38]. Nucleotide diversity (π) values were calculated using the Jukes and Cantor method and DNA Polymorphism values for whole mtDNA were calculated with window length of 50 sites and step size of 25 base pairs (Figure S3) using DNAsp v.6.12.03 (Rozas et al., Barcellona, Spain) [39]. Ratio of non-synonymous vs. synonymous mutations was calculated using mtDNA GeneSyn v1.0 (Pavesi et al., Milan, Italy) [40].

### 4.3. Phylogenetic Analysis

A maximum parsimony (MP) tree for *cytb* variation was manually built to confirm main haplogroup mutations and later confirmed by using Molecular Evolutionary Genetics Analysis X, MEGA v.11.0.13 (Tamura et al., Tokyo, Japan; Philadelphia, PA, USA) [35]. An MP tree was built using the GTR model (8 gamma-distributed categories) with 1000 bootstraps (extensive SPR method) to obtain likelihood values for the main haplogroups. The tree was rooted using *Panthera pardus* (KP001507), *Panthera onca* (NC022842), and two *Panthera tigris* (NC010642, AF053054). Given the publication of at least seven different mtDNA haplogroups nomenclatures from the literature [3,13,20,21,22,41,42,43] and two for cave lions [8,9], we tried to standardise the nomenclatures and A to F were chosen for *P. leo*, S for *P. spelaea*, and X for *P. atrox.* All haplogroups (including a conversion table from other works) can be found in Table S3. The haplotype network was constructed using Fitchi v 1.1.4 [44]. The script was run using the following flags: -haploid; -p [A–X populations]. The coding region parsimony tree was built using MEGA v.11.0.13 (Tamura et al., Tokyo, Japan; Philadelphia, PA, USA) (Figure 2); it can be seen in greater detail in Figure S2 and was built by programming a script for the software mtPhyl v 5.003 (Eltsov & Volodko, Novosibirsk, Russia) [45] (modified files are available on request); indels and control region were not considered for this analysis due to the large gaps present in ancient samples compared to the reference sequence.

### 4.4. Molecular Clock and Tree Dating

For age estimations we used a combination of two methods and calculated coalescence ages for both *cytb* and the whole mtDNA. Firstly, we estimated ML values using the BaseML package present in PAMLX v1.3.1 (Xu & Yang, Beijing, China) [46]. These were performed assuming a HKY85 mutation model [47], 32 categories of gamma-distributed rates (plus invariant sites) and either first, second, and third codon as three partitions [48] or 17 partitions (13 for protein coding genes, 1 for tRNAs, 1 for each of the two rRNA gene, and 1 for intergenic regions) using the previously obtained maximum parsimony tree [49]. In both cases, ML mutational distances were converted into years by assuming an estimated split time between *P. pardus* and *P. leo* at 2.96 Mya (95% CI: 1.4–4.5 Mya) [50]. This standard molecular approach does not include the error of a calibration point using the debated and often misattributed *Panthera fossilis* (or *Panthera spelaea fossilis*) calibration point [15,25,49,51,52,53], which has previously been used for root calibration. A mutation rate for *cytb* and the whole mtDNA was then calculated from the ML dates; these were 0.01797 and 0.01327 s/s/l/m (or one mutation every 53,522 and 4854 years, respectively).

Bayesian estimations were then performed using the software Bayesian Evolutionary Analysis Sampling Trees (BEAST) v.2.7.5 (Bouckaert et al., Auckland, New Zealand) [54] under the HKY nucleotide substitution model (8 gamma-distributed rates plus 0.5 proportion invariant sites) with a strict clock (uniform using ML calculated rates) [49,55]. Fossil dates for all *atrox* and *spelaea* samples were set as priors with the addition of the following node age constraints: S (0.11–0.1354 Mya); X (0.0747–0.0876 Mya); SX (0.145–0.185 Mya); and ASX (0.52–2.91 Mya). The different outgroup splits were derived from the literature (uniform distribution with standard deviation) [6,8,9,49]. The MCMC chain length was set at 100 million iterations to increase the posterior values and samples were drawn every 1000 steps with 10 million steps discarded as burn-in. We constructed Bayesian skyline plots (BSPs) using the output tree file and a stepwise constant.

Haplogroup expansion maps were built using Surfer^®^ v 9.11 (La et al., Golden, CO, USA) using the point kriging method (Figure S9) and overlayed manually on a world map.

### 4.5. Genotyping and Population Structure

A set of seven microsatellite markers, chosen among those utilised by Bertola et al., [13], was used for all the analysis: FCA075, FCA097, FCA126, FCA144, FCA208, FCA211, and FCA275. Sequences, annealing temperatures, and PIC values of each pair of primers used in this work are reported in Table S3. PCR amplification of microsatellite loci was prepared using 0.2 μL of Taq DNA polymerase (MyTaq, Meridian Bioscience, Cincinnati, OH, USA) in 10 μL reaction with final conditions as follows: 5X PCR buffer, 1 μL of primer mix (forward and reverse), and 1 μL of genomic DNA template. PCR amplification was conducted in an Eppendorf AG 22331 Thermal Cycler as follows: 95 °C for 3 min, 30 cycles for 15 s at 95 °C, 15 s at the optimum Ta, and 30 s at 72 °C, followed by extension at 72 °C for 10 min.

Amplification products were analysed by an automatic sequencer with capillary technology; genotypic data were obtained by using Peak Scanner v.2.0 (ThermoFisher Scientific, Waltham, MA, USA). Genotypic data from other populations used as comparison (Table S5) were obtained from the database published by Bertola et al., [56].

We then calculated the allelic frequencies, the expected and observed heterozygosity at each locus for all populations, and the *F*_ST_ statistics according to the estimate of Weir and Cockerham [17] to determine the genetic diversity within and between them (Table S6). Genetix v.4.05 (Belkhir et al., Montpellier, France) [16] was used to estimate the divergence between the populations under study by means of correspondence factor analysis. The software structure v.2.3.4 (Pritchard, Stanford, CA, USA) was used to investigate the genetic composition of the population; 10 runs for each *K* were run from *K* = 1 to *K* = 12 using Evanno’s Δ*K* method [18]. In Table S7 we report the probability that a given population may have originated from the same ancestral population. Finally, using v.1.1.2 (Rosenberg, Stanford, CA, USA) and distruct v.1.1 (Rosenberg, Stanford, CA, USA) on the structure results, we obtained the barplot of the average run for each *K* value (Figure 6).

## 5. Conclusions

Among the big charismatic carnivores, the tiger (*Panthera tigris*) and the lion have seen the largest reductions in their respective ranges, with 95% and 94%, respectively [56], due to anthropogenic impacts. The main consequence of this is that gene flow is made difficult because of isolation due to the physical distances involved; this can be especially important in the case of lions, which live in large family groups and, therefore, the phenomenon of inbreeding can be particularly evident. 

Henschel and coworkers [57] brought to light that only approximately 400 lions remain in West Africa, highlighting how the situation is critical due to the geographical isolation of these populations, which present a genetic peculiarity that would make them “incompatible” with individuals from the rest of the continent. This proves the fact that simply moving lions from distant areas to areas in need of restocking is not the solution.

Many studies have focused their attention on lion mtDNA, without fully exploiting its phylogenetic potential. These studies mainly assessed *cytb* sequences and each one used different nomenclatures to refer to the same haplogroups [8,9,13,15,17,45,46,47,48]. Taking these data, together with the available whole mtDNAs, we constructed a complete and detailed phylogeny for three lion species and reconstructed their genetic history and relationships, and presented a standardised nomenclature. We found that *cytb* alone, as far as lions are concerned, is informative enough to correctly classify individuals into six main *Panthera leo* haplogroups, distinguish the two modern lion subspecies from one another, and discern modern lions from Eurasian and American cave lions. The addition of whole mtDNA samples helped with an even more precise phylogenetic reconstruction, though in smaller numbers and spanning fewer haplogroups.

We have found a species/subspecies geographic specificity within major mitogenome haplogroups. These distinctions have emerged at various temporal and spatial instances. Leveraging estimated radiation times, we have constructed a unified framework to hypothesize the overarching patterns governing the geographical and temporal dispersion of modern lion populations across Africa and Asia and the spread of their cave ancestors in Eurasia and North America. This phylogenetic assessment will be beneficial for lion conservation, by providing a better classification of the various subspecies/lineages.

## Data Availability

Samples were deposited in Genbank under the following accession numbers: 15 modern lions, KX110059–KX110073.

## Supplementary Materials

The following supporting information can be downloaded at: https://www.mdpi.com/article/10.3390/ijms25105193/s1.
