# Supplementary material for: From Caves to the Savannah, the Mitogenome History of Modern Lions (Panthera leo) and Their Ancestors
Source: Int J Mol Sci. 2024 May 10;25(10):5193. doi: 10.3390/ijms25105193 (PMC11121052; doi:10.3390/ijms25105193)
Supplement: Supplementary file 1 [file ijms-25-05193-s001.zip › Broggini et al IJMS - Supplementary material.pdf]

International Journal of
Molecular Sciences

MDPI

Article

From Caves to the Savannah, the Mitogenome History of Modern Lions (*Panthera leo*) and Their Ancestors

Camilla Broggini [1,†], Marta Cavallini [2,†], Isabella Vanetti [2], Jackie Abell [3], Giorgio Binelli [2] and Gianluca Lombardo [2,*]

[1] Wildlife Research Unit (UIRCP-UCO), University of Cordoba, 14071 Córdoba, Spain; z62brbrc@uco.es
[2] Department of Biotechnology and Life Sciences (DBSV), University of Insubria, 21100 Varese, Italy; m.cavallini1@uninsubria.it (M.C.); isabella.vanetti@uninsubria.it (I.V.); giorgio.binelli@uninsubria.it (G.B.)
[3] Centre for Agroecology, Water and Resilience, Coventry University, Coventry CV8 3LG, UK; jackie.abell2@coventry.ac.uk
* Correspondence: gianluca.lombardo@uninsubria.it
† These authors contributed equally to this work.

Citation: Broggini, C.; Cavallini, M.; Vanetti, I.; Abell, J.; Binelli, G.; Lombardo, G. From Caves to the Savannah, the Mitogenome History of Modern Lions (*Panthera leo*) and Their Ancestors. *Int. J. Mol. Sci.* **2024**, *25*, 5193. https://doi.org/10.3390/ijms25105193

Academic Editor: Brad Freking

Received: 16 April 2024
Revised: 6 May 2024
Accepted: 8 May 2024
Published: 10 May 2024

Copyright: © 2024 by the authors. Submitted for possible open access publication under the terms and conditions of the Creative Commons Attribution (CC BY) license (https://creativecommons.org/licenses/by/4.0/).

1. Supplementary Figures

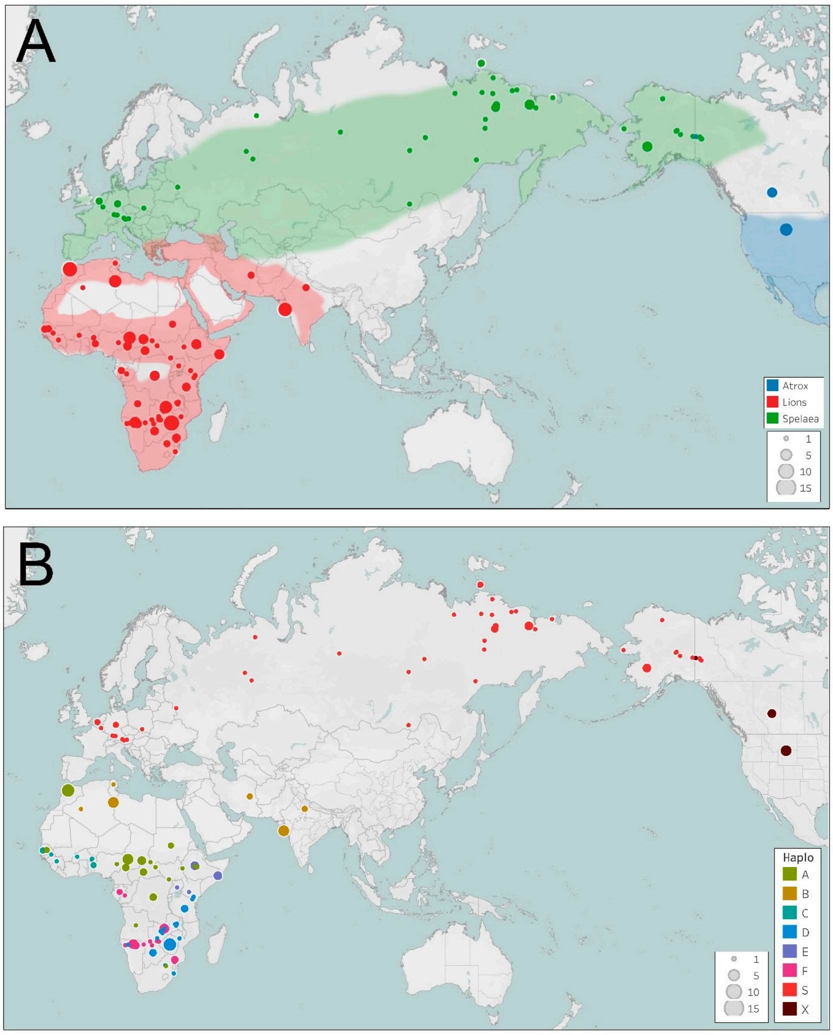

Figure S1. Distribution map of the three lion species. (A) Shaded areas indicate distribution ranges of the species while circles indicate the sampling locations (when available from Genbank). (B)

Haplogroup distribution of samples used in this study. Colours indicate species in A and haplogroups in B. Circle sizes are representative of the number of samples. Distribution map modified from (Nicholson et al., 2023) and (Barnett et al., 2016).

Figure S2. Detailed maximum parsimony phylogeny of lion mitogenomes. This tree was built using the entire mitogenome coding-region of the available lion mitogenomes and was rooted using *P. pardus* (KP001507, Bertola et al., 2016). Main haplogroup and sub-haplogroup affiliations are shown. Sub-haplogroups were named only when encompassing at least two haplotypes. Species/Subspecies affiliations are according to the colours in the legend. Mutations, relative to the lion reference sequence (NC_028302), are transitions unless a base is explicitly indicated. Suffixes indicate transversions (to A, G, C, or T). Reversions are marked with "@" and recurring mutations are underlined. Synonymous mutations are highlighted in green and non-synonymous mutations in yellow. Heteroplasmic positions are shown below sample names.

EXCEL FILE

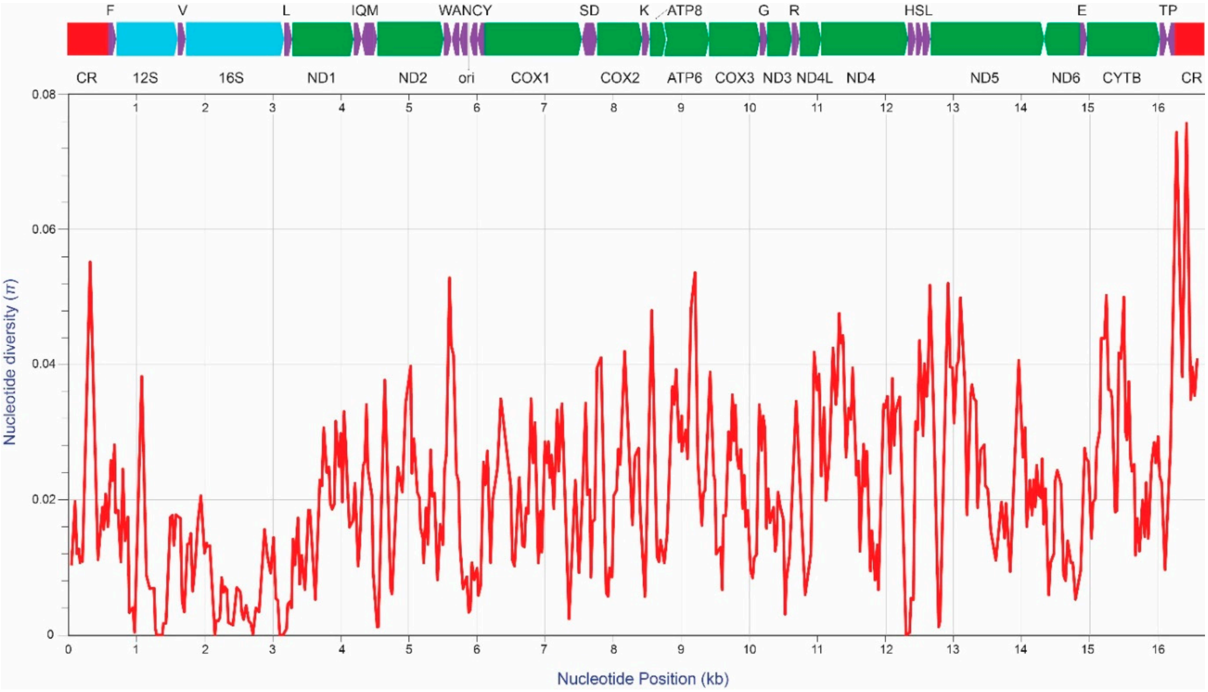

Figure S3. Nucleotide diversity in *Panthera leo* mitogenome loci. The linearised gene map of modern lions is shown above. Protein coding genes are in green, rRNA genes in blue, tRNA genes in purple and the D-loop in red. Arrow direction indicate the direction of gene transcription. Nucleotide diversity (continuous red line) of the entire mitogenome (50 bp windows, 25 pair steps) is shown below.

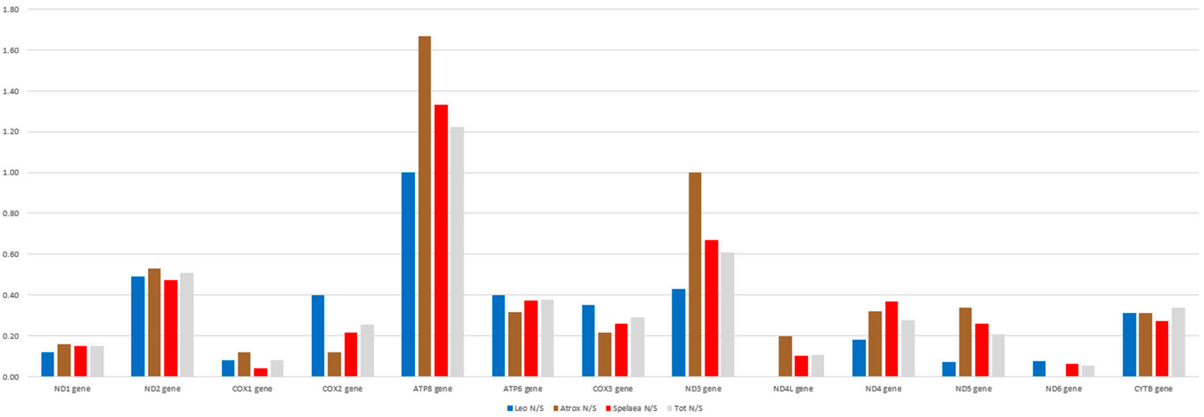

Figure S4. Ratio of non-synonymous vs synonymous mutations. Colours indicate: *Panthera leo* (blue), *P. spelaea* (red), *P. atrox* (brown) and all lions (grey). A N/S ratio <1 roughly indicates purifying selection, while a ratio of >1 indicates positive selection. This bar chart was constructed using mtDNA GeneSyn v1.0.

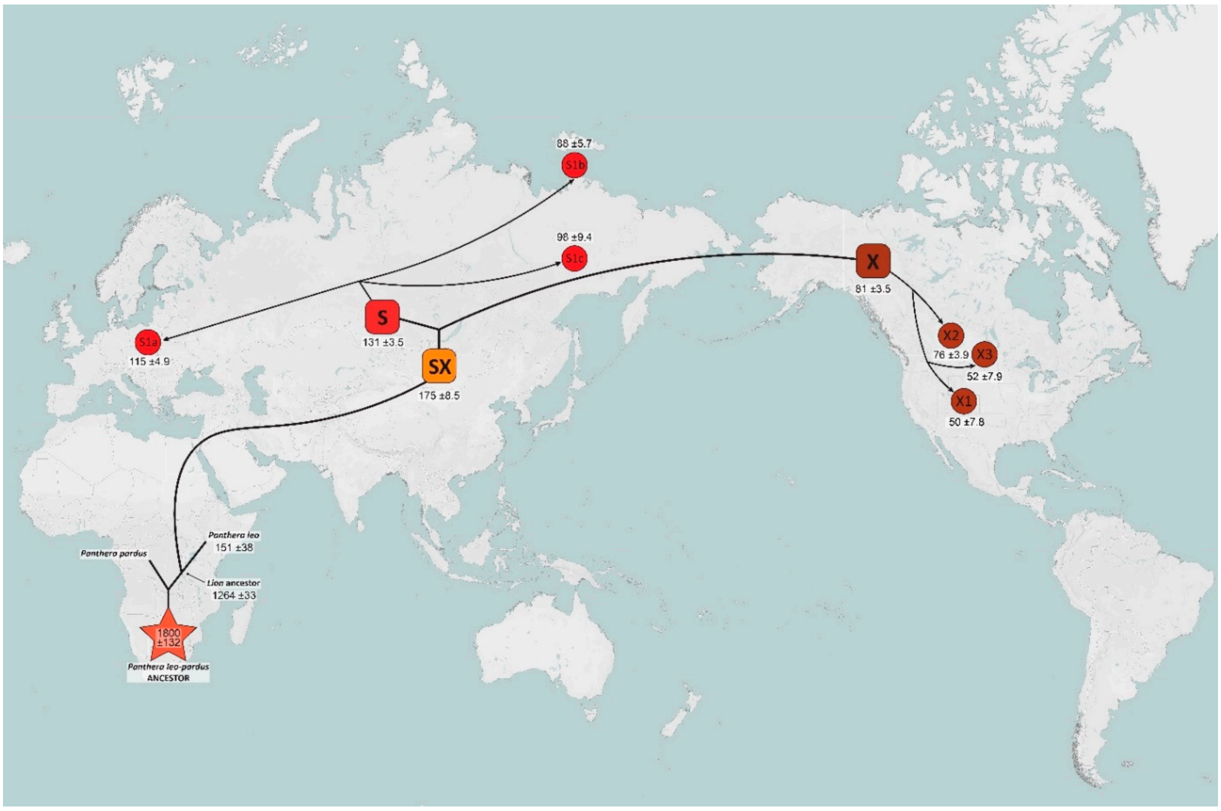

Figure S5. Cave lion species spread through time. Map is based on *cytb* divergence times obtained with ML molecular clock and Bayesian analysis. Squares are haplogroups and circles are sub-haplogroups. The colours are consistent with the Figures in the main text. Lines indicate haplogroup expansions. Numbers above or below haplogroups indicate the coalescence times in thousands of years ago (kya) with their respective standard deviation. Numbers at nodes indicate separation times between lineages. The red star indicated the ancestral lion mitogenome (LiAM), or mitochondrial eve.

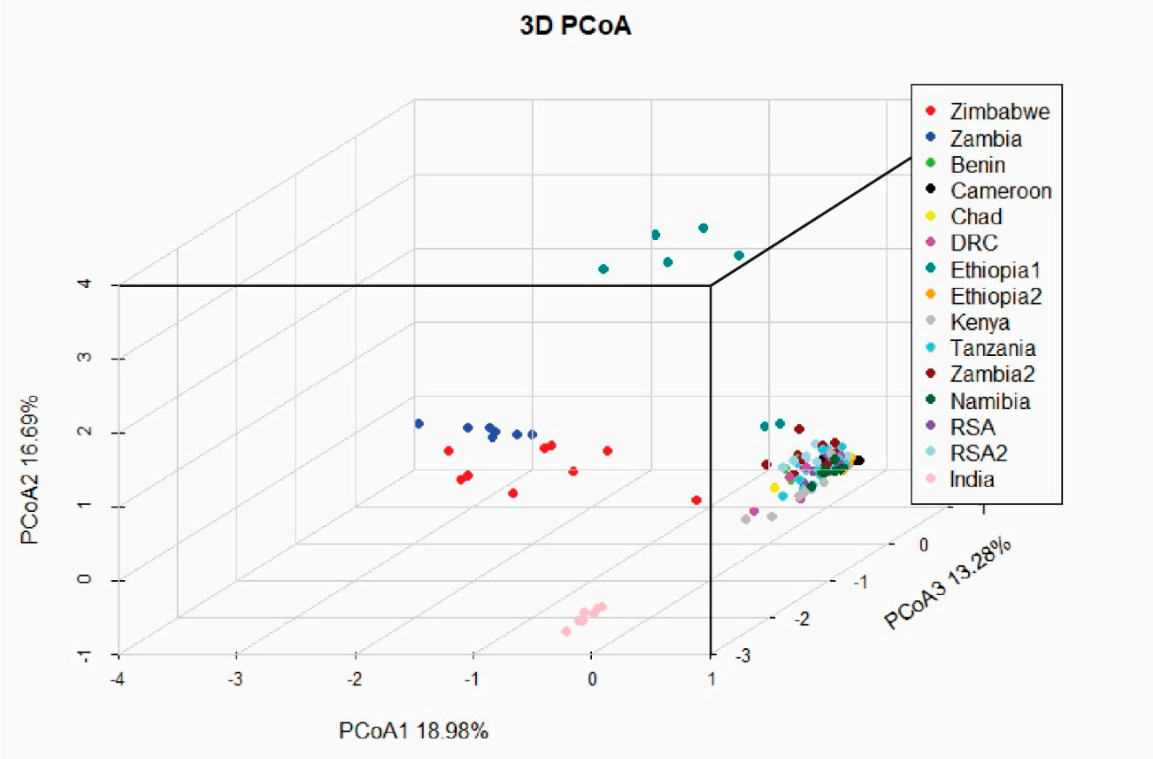

Figure S6. Factorial analysis of correspondences. Each lion is represented by a point in three-dimensional space. Our populations

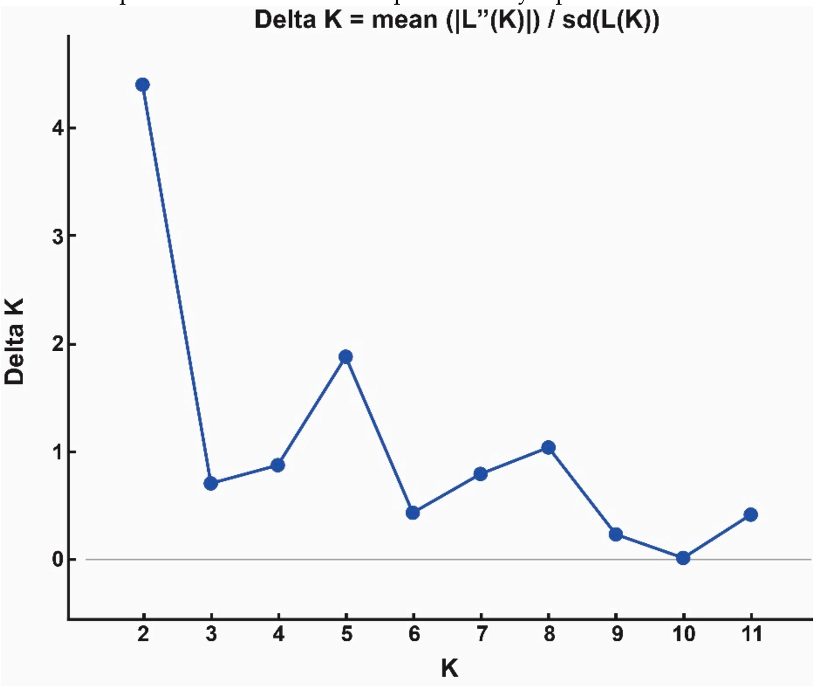

are grouped at the top left while the others African populations are on the right. India is the pink group at the bottom.

Figure S7. Most likely ΔK value. Estimate of the most probable number for *K*, based on the method of Evanno *et al*. (2005). The highest value of Δ*K* corresponds to the most probable value of *K*.

Figure S8. Map of the *Panthera leo* mitogenome. This map refers to the reference sequence (PLE, mitogenome ID #1, NC028302) obtained and modified from CGview Server (Grant et al., 2023). Genes are represented as blocks of different colours with direction of transcription shown by arrows. CDS indicates the 13 protein-coding genes. tRNA genes are labelled according to IUPAC amino acid code. GC content is plotted in black using a sliding window, as the deviation from the average GC content of the entire sequence. Positive and negative GC skews are relative to the average GC content of the entire sequence. The CR is 1167 bps in length, and maps between the *MT-TP* and *MT-TF* genes. The heavy strand base composition comprises A-5310 (31.9%), T-4520 (27.2%), C-4395 (26.4%) and G-2395 (14.4%), with an A+T (59.1%) content higher than the G+C content.

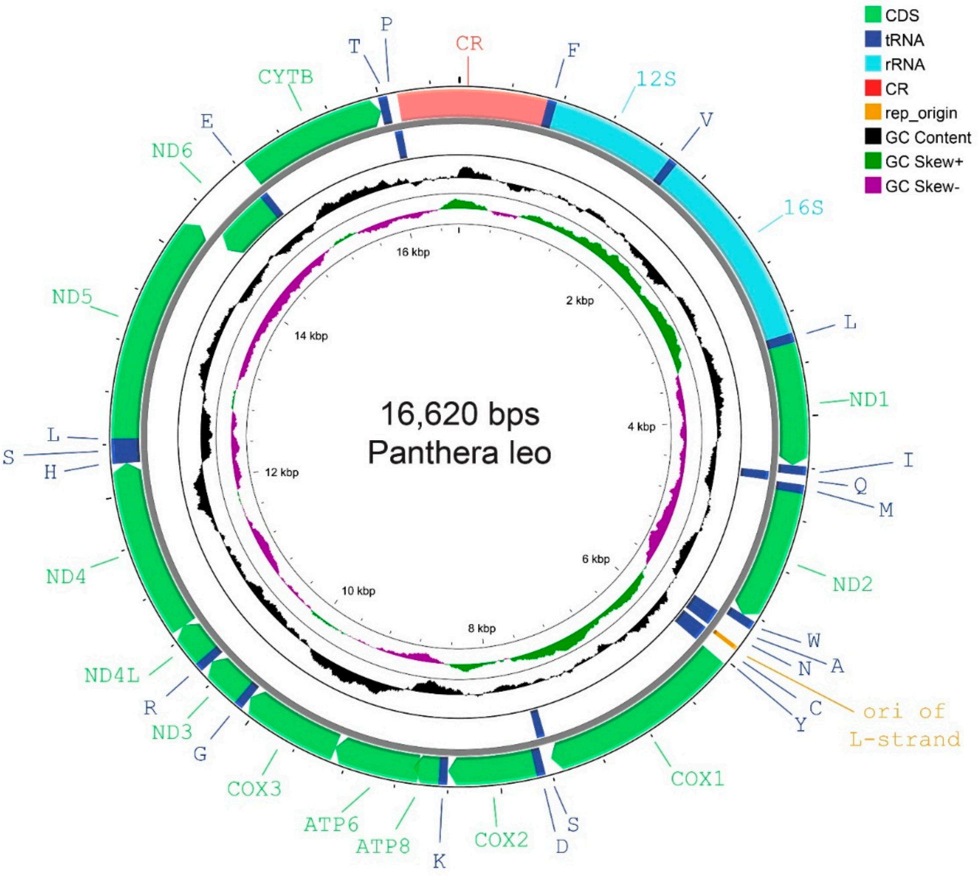

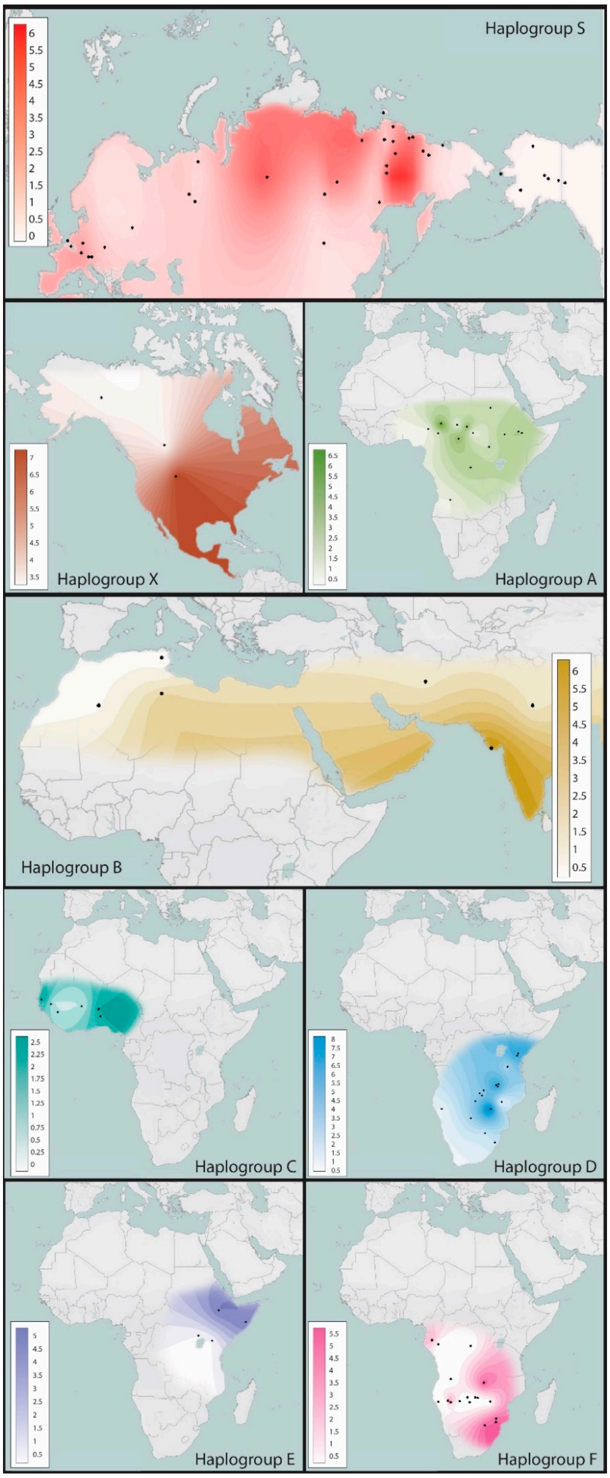

Figure S9. Spatial frequency distribution maps of main lion haplogroups. Dots indicate the geographical locations of the sampled individuals. Colour scale indicates frequency of haplogroup in the given map. Haplogroup S represents *Panthera spelaea*, haplogroup X is *Panthera atrox* and all other haplogroups encompass *Panthera leo*. Maps were generated with Surfer® program (v 9.11, Golden Software, Inc., Golden, CO, USA, www.goldensoftware.com/).

2. Supplementary Tables

Table S1. Nucleotide diversity heatmaps (%) of *cytb* within and between haplogroups (A) and species (B). Intragroup nucleotide diversities (π) are on the diagonal. Standard deviation is reported in brackets. The colour scale indicates relationship, green is genetically close while red is more distant.

Panthera leo Haplogroups — S1A	A	B	C	D	E	F
A	0.053 ± 0.01	0.535 ± 0.10	0.307 ± 0.07	0.894 ± 0.13	0.918 ± 0.21	1.118 ± 0.15
B	-	0.160 ± 0.07	0.433 ± 0.11	0.798 ± 0.13	0.859 ± 0.21	1.091 ± 0.16
C	-	-	0.029 ± 0.02	0.840 ± 0.17	0.816 ± 0.24	1.046 ± 0.18
D	-	-	-	0.116 ± 0.02	0.570 ± 0.11	0.789 ± 0.09
E	-	-	-	-	0.142 ± 0.03	0.771 ± 0.13
F	-	-	-	-	-	0.328 ± 0.04

S1B	*P. leo*	*P. spelaea*	*P. atrox*	All cats
P. leo	0.598 ± 0.02	4.452 ± 0.20	4.873 ± 0.46	1.856 ± 0.15
P. spelaea	-	0.358 ± 0.04	1.744 ± 0.17	3.395 ± 0.19
P. atrox	-	-	0.270 ± 0.04	4.007 ± 0.35
All cats	-	-	-	2.441 ± 0.15

Table S2. Coalescence age estimates of haplogroups using *cytb* and whole mtDNA. Bayesian age estimates for lion haplogroups and sub-haplogroups. Estimates are based on the MP phylogeny (227 *cytb* sequences, 1040 bp and 61 whole mitogenomes, 16,620 bp) of Figures 1 and 2.

EXCEL FILE

Table S3. Lion samples analysed for mtDNA variation. * indicates no specific geographical information aside from country

EXCEL FILE

Table S4. Primers used for amplification of fragments and respective annealing temperatures (T_a).

Primer name	Sequence (5′-3′)	T_a (°C)	PIC
FCA075_TMR	ATGCTAATCAGTGGCATTTGG	57	0.904
FCA075_R	GAACAAAAATTCCAGACGTGC	57	
FCA097_FAM	TAATGTTCAACTTGAATTGCTTCC	58	0.873
FCA097_R	GAACAGTAGTTTGCCCATACAGG	63	
FCA126_TAMRA	GCCCCTGATACCCTGAATG	59	0.826
FCA126_R	CTATCCTTGCTGGCTGAAGG	60	
FCA144_HEX	GGAAATCCTGGAAACTTCTGC	59	0.698
FCA144_R	CCCGGCAAAATTATGAAGG	55	
FCA208_HEX	TCAGGGTTCAAAAAAAGAAAAA	53	0.825
FCA208_R	CAAAGCACCAGCTTAGAAGTCA	60	
FCA211_ROX	TGTAGAACATAATGCCTCAGCC	60	0.770
FCA211_R	TCTTGAACCTATTTCCCCACA	57	
FCA275_FAM	TTGGCTGCCCAGTTTTAGTT	56	0.803
FCA275_R	ACGAAGGGGCAGGACTATCT	60	

Table S5. Estimation of genetic variability. H_e = expected heterozygosity, H_o = observed heterozygosity, $\bar{x}$ = mean number of alleles per locus.

Population	H_e	H_o	$\bar{x}$	F_{IS}	Avg alleles/ locus
Zimbabwe	0.70	0.67	5.57	0.12	5.57
Zambia	0.59	0.49	4.83	0.27	4.83
Benin	0.63	0.83	3.57	-0.22	3.57
Cameroon	0.61	0.71	4.43	-0.12	4.43
Chad	0.59	0.63	4.00	0.09	4.00
DRC	0.65	0.81	5.00	-0.17	5.00
Ethiopia1	0.51	0.73	3.29	-0.27	3.29
Ethiopia2	0.46	0.58	2.33	-0.14	2.33
Kenya	0.55	0.59	3.00	-0.01	3.00
Tanzania	0.62	0.63	5.00	0.01	5.00
Zambia	0.70	0.71	5.43	0.04	5.43
Namibia	0.50	0.52	3.29	0.01	3.29
RSA-A	0.58	0.54	4.00	0.12	4.00
RSA-B	0.62	0.59	4.00	0.10	4.00
India	0.14	0.17	1.57	-0.12	1.57
Mean values	0.56	0.61	3.95	-0.02	3.95

Table S6. Estimates of F_{ST} between pairs of populations. All values are significantly different from zero.

	Zambia	Benin	Cameroon	Chad	DRC	Ethiopia1	Ethiopia2	Kenya	Tanzania	Zambia	Namibia	RSA-A	RSA-B	India
Zimbabwe	0.12	0.23	0.27	0.25	0.23	0.25	0.32	0.31	0.28	0.20	0.36	0.26	0.27	0.52
Zambia		0.23	0.32	0.26	0.28	0.22	0.36	0.34	0.32	0.25	0.40	0.31	0.30	0.62
Benin			0.20	0.18	0.19	0.20	0.30	0.22	0.19	0.10	0.27	0.21	0.23	0.61
Cameroon				0.09	0.11	0.27	0.27	0.27	0.23	0.16	0.29	0.21	0.24	0.58
Chad					0.13	0.20	0.23	0.18	0.19	0.15	0.22	0.15	0.23	0.64
DRC						0.21	0.25	0.26	0.19	0.09	0.24	0.16	0.22	0.56
Ethiopia1							0.35	0.29	0.29	0.15	0.27	0.27	0.25	0.65
Ethiopia2								0.31	0.28	0.19	0.33	0.27	0.24	0.71
Kenya									0.14	0.21	0.37	0.27	0.28	0.58
Tanzania										0.10	0.30	0.17	0.26	0.53
Zambia											0.23	0.13	0.15	0.55
Namibia												0.25	0.26	0.63
RSA-A													0.22	0.61
RSA-B														0.57

Table S7. Percentages of the studied populations of belonging to each inferred cluster. Values above 50% are highlighted in bold. NO ADMIX (*K* = 5). Hg = Haplogroup. Zimbabwe and Zambia 1 are populations from this study while all the others are from literature (Bertola et al., 2011).

Given Pop	Inferred Clusters					Number of Individuals	MtDNA Hg equivalent
	1	2	3	4	5		
Zimbabwe	0.014	0.013	0.025	0.000	0.948	9	D
Zambia	0.000	0.000	0.000	0.000	1.000	7	D
Benin	0.402	0.134	0.464	0.000	0.000	5	-
Cameroon	0.000	0.000	1.000	0.000	0.000	12	A
Chad	0.035	0.026	0.940	0.000	0.000	4	A
DRC	0.000	0.025	0.975	0.000	0.000	7	A
Ethiopia1	0.044	0.098	0.097	0.667	0.094	15	B
Ethiopia2	0.005	0.097	0.898	0.000	0.000	4	A
Kenya	0.999	0.000	0.001	0.000	0.000	7	E
Tanzania	0.957	0.034	0.009	0.000	0.000	20	E
Zambia 2	0.438	0.321	0.237	0.000	0.004	9	-
Namibia	0.000	1.000	0.000	0.000	0.000	10	F
RSA-A	0.077	0.922	0.001	0.000	0.000	10	F
RSA-B	0.000	0.999	0.000	0.000	0.000	10	F
India	0.000	0.000	0.000	1.000	0.000	10	B

Table S8. Information about the 17 sampled lions. Their date of birth, sex, sampling site and sampling date are reported together with their Genbank accession code.

Sample ID	Sampling Date	Sampling Site	Sex	Date of Birth	Accession N°
KE	07/10/2015	Zimbabwe	F	31/10/2004	KX110060
AT1	06/10/2015	Zimbabwe	F	20/01/2011	KX110066
K4	06/10/2015	Zimbabwe	F	04/10/2011	KX110061
AS5	09/10/2015	Zimbabwe	M	04/11/2011	KX110072
AG	10/10/2015	Zimbabwe	F	---	---
LU	10/10/2015	Zimbabwe	M	19/01/2007	KX110059
AF	03/10/2015	Zimbabwe	M	---	KX110073
AM	05/10/2015	Zimbabwe	M	---	KX110067
KY	06/10/2015	Zimbabwe	F	30/04/2009	KX110062
M+	09/10/2015	Zimbabwe	M	---	KX110071
TI	16/10/2015	Zambia	F	06/05/2014	KX110068
NA	16/10/2015	Zambia	F	---	KX110063
SE	18/10/2015	Zambia	M	20/03/2014	---
NE	16/10/2015	Zambia	F	---	KX110065
SA	17/10/2015	Zambia	F	20/03/2014	KX110069
SM	19/10/2015	Zambia	F	20/03/2014	KX110070
X	20/10/2015	Zambia	---	---	KX110064

Table S9. Primers used for amplification of the 718 bp *cytochrome b* fragment and their annealing temperatures (T_a).

Primer name	Sequence (5′-3′)	T_a (°C)
2F	GTGGGGCCAAATATCCTTTT	52
4R	TTTTTGGTTTACAAGACCAAGGTA	52

Disclaimer/Publisher's Note: The statements, opinions and data contained in all publications are solely those of the individual author(s) and contributor(s) and not of MDPI and/or the editor(s). MDPI and/or the editor(s) disclaim responsibility for any injury to people or property resulting from any ideas, methods, instructions or products referred to in the content.
