# Supplementary figures and images for: From Caves to the Savannah, the Mitogenome History of Modern Lions (Panthera leo) and Their Ancestors
Source: Int J Mol Sci. 2024 May 10;25(10):5193. doi: 10.3390/ijms25105193 (PMC11121052; doi:10.3390/ijms25105193)

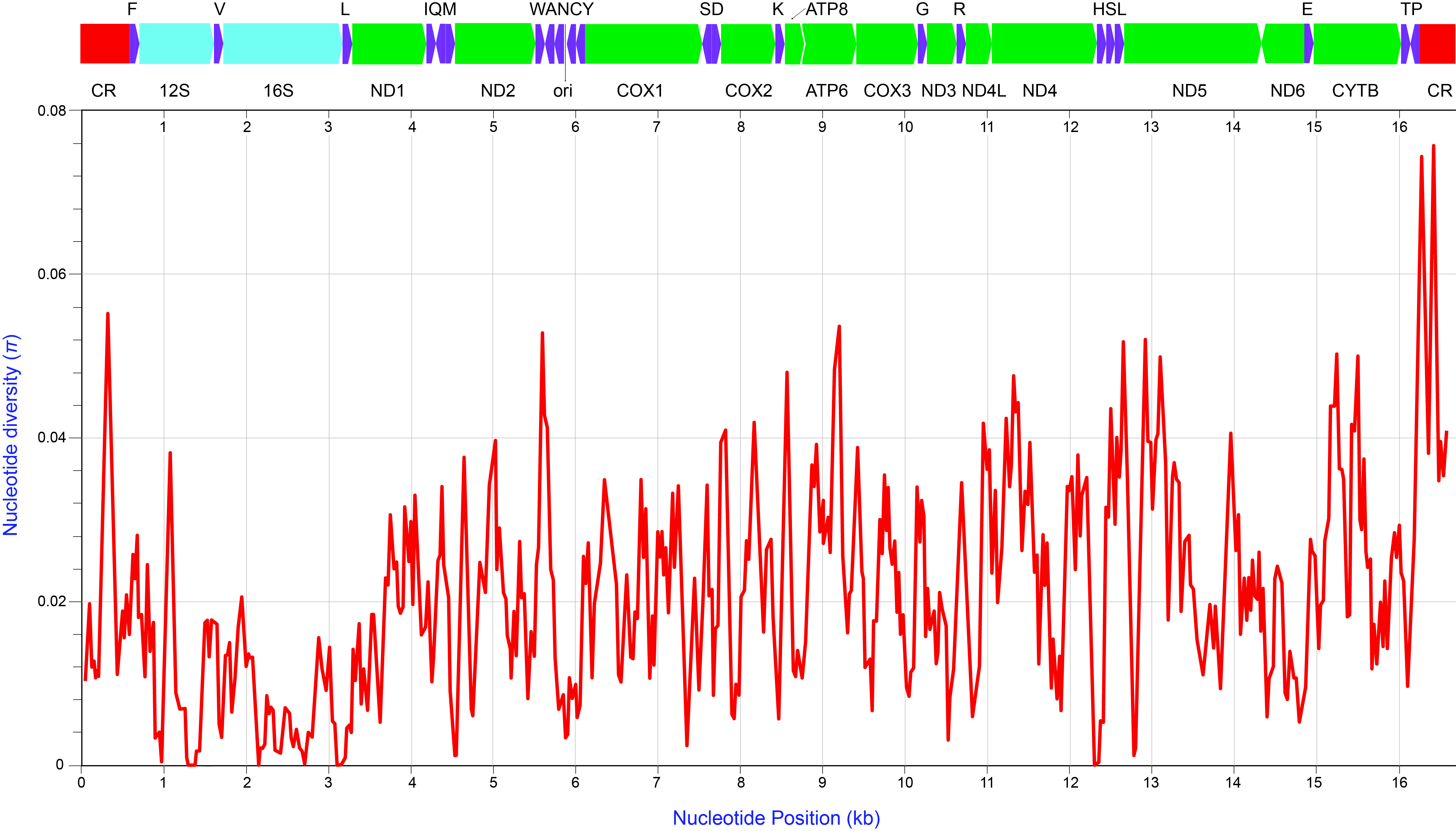

Supplement: Supplementary file 1 [file ijms-25-05193-s001.zip › Figure S3.jpg]

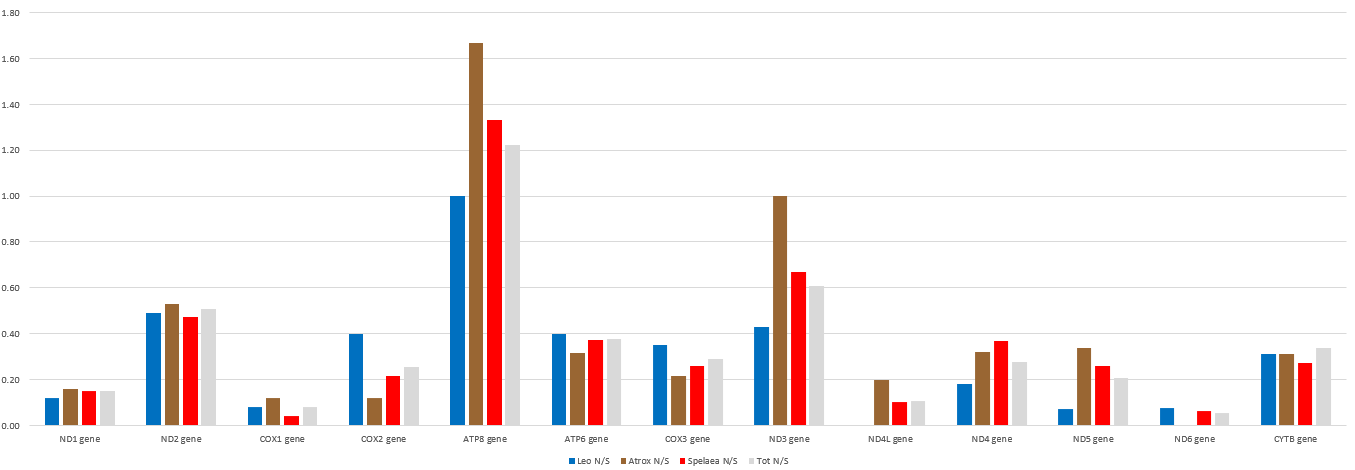

Supplement: Supplementary file 1 [file ijms-25-05193-s001.zip › Figure S4.png]

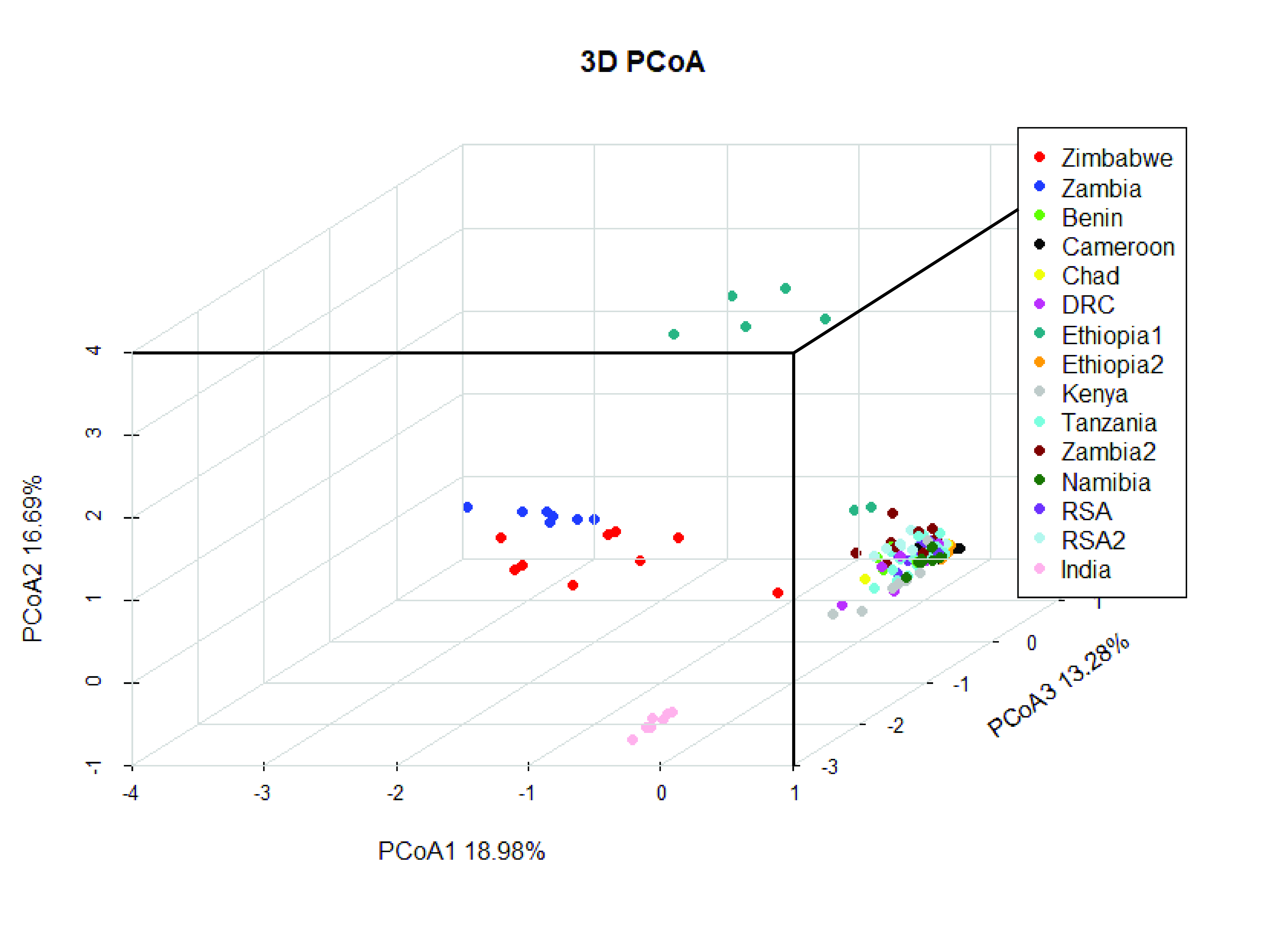

Supplement: Supplementary file 1 [file ijms-25-05193-s001.zip › Figure S6.jpg]

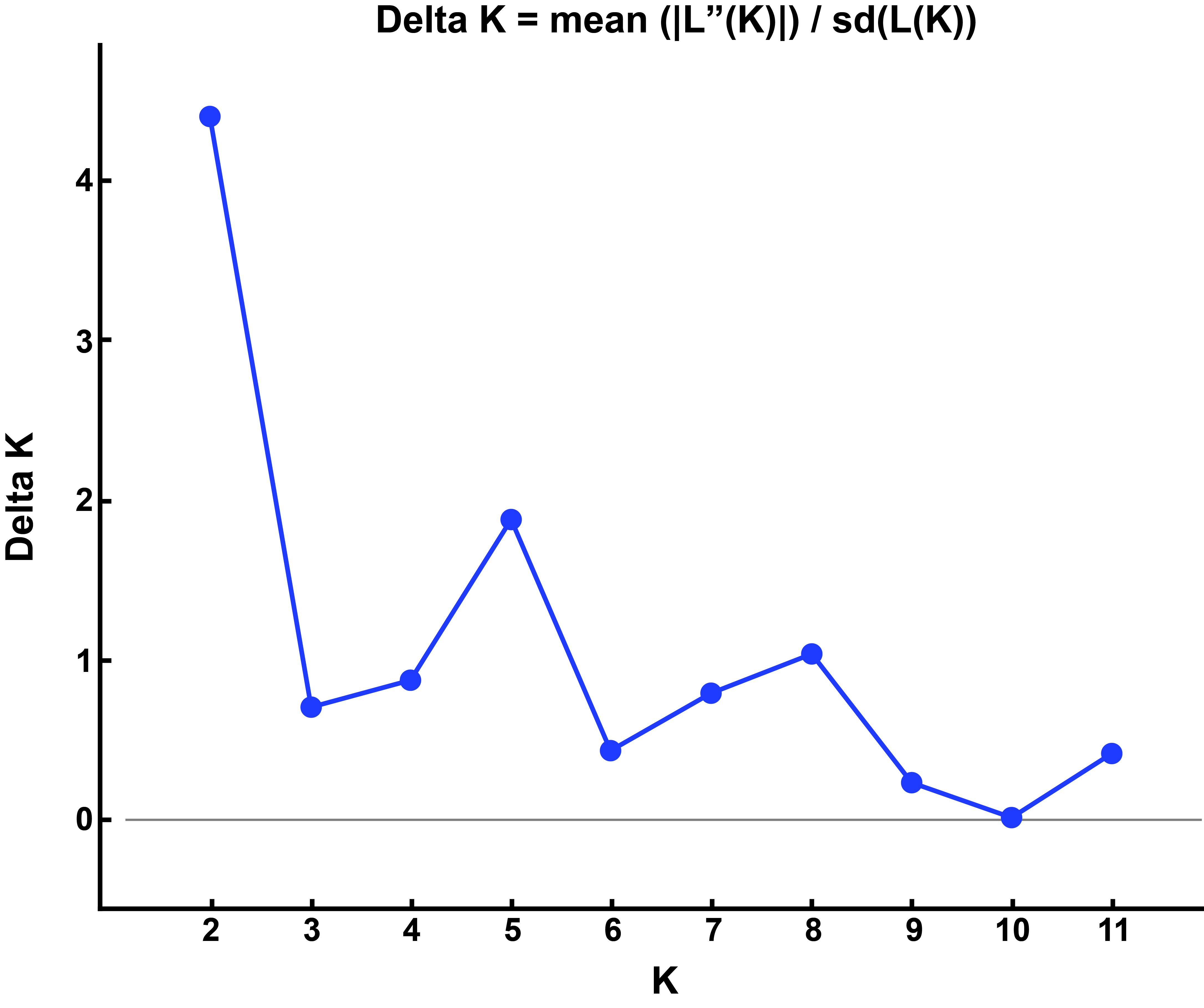

Supplement: Supplementary file 1 [file ijms-25-05193-s001.zip › Figure S7.jpg]

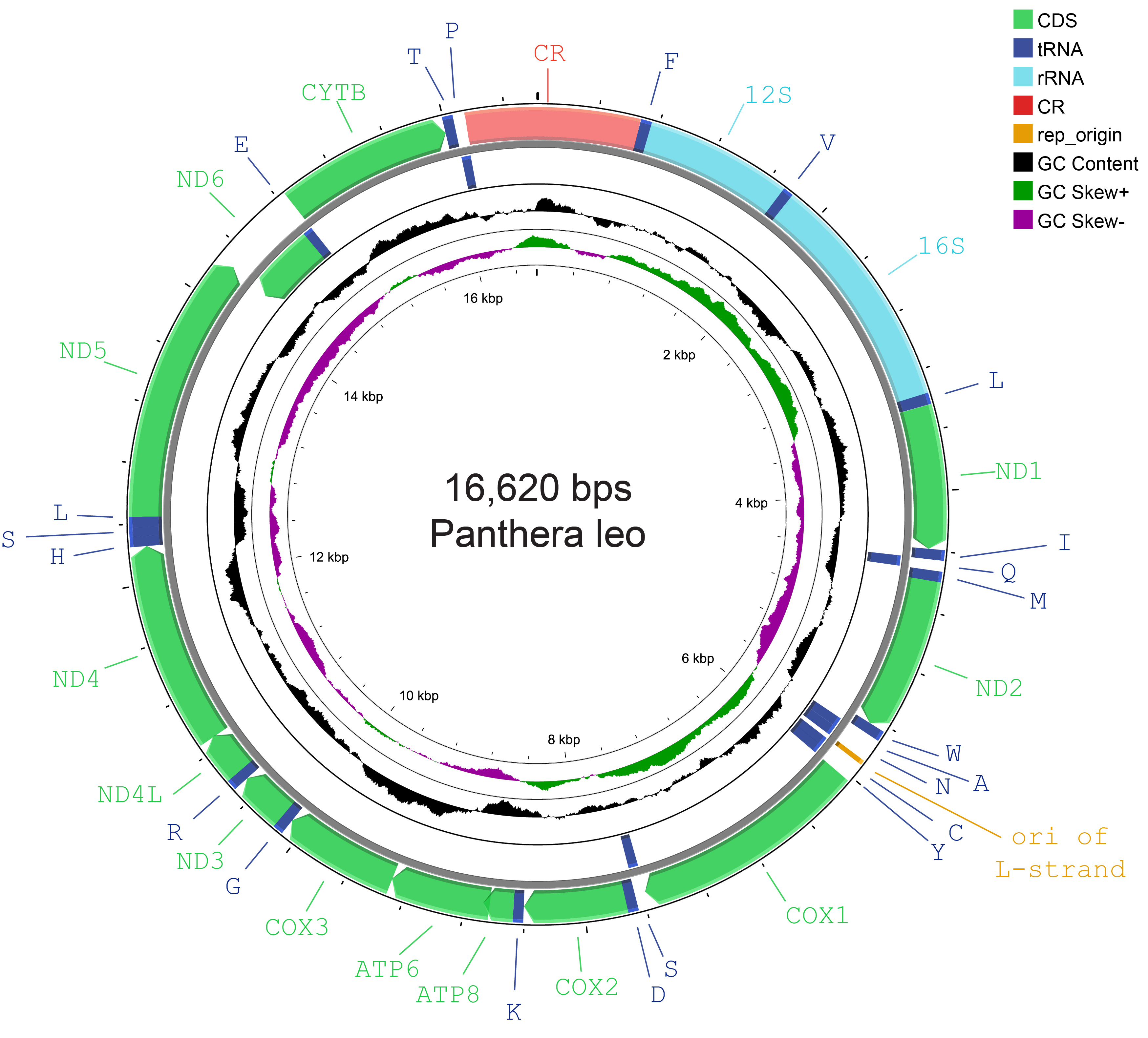

Supplement: Supplementary file 1 [file ijms-25-05193-s001.zip › Figure S8.jpg]

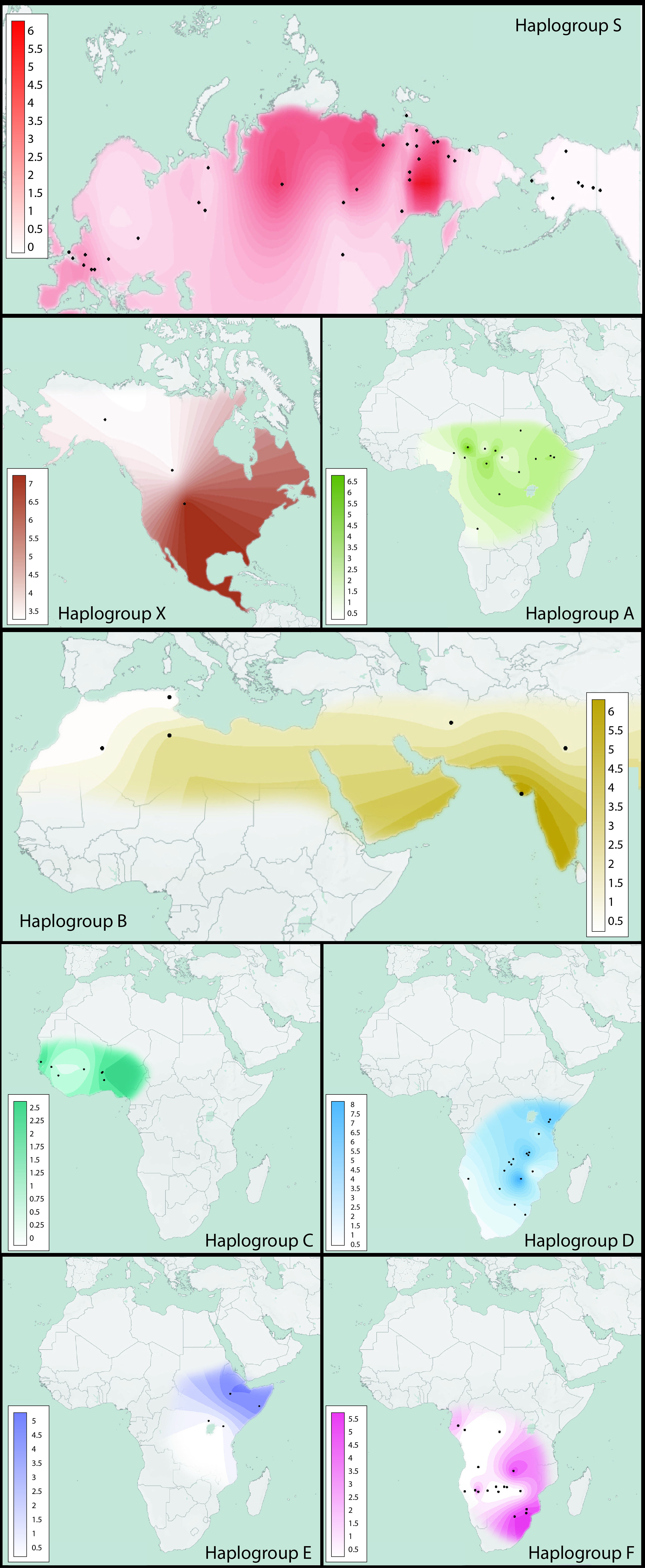

Supplement: Supplementary file 1 [file ijms-25-05193-s001.zip › Figure S9.jpg]
